# Effect of Burned Multi-Crop Ashes on Faba Bean-Development Parameters

**DOI:** 10.3390/plants13162182

**Published:** 2024-08-07

**Authors:** Rita Petlickaitė, Kęstutis Romaneckas, Aušra Sinkevičienė, Marius Praspaliauskas, Algirdas Jasinskas

**Affiliations:** 1 Laboratory of Heat-Equipment Research and Testing, Lithuanian Energy Institute, Breslaujos Str. 3, LT-44403 Kaunas, Lithuania; marius.praspaliauskas@lei.lt; 2 Department of Agroecosystems and Soil Sciences, Faculty of Agronomy, Agriculture Academy, Vytautas Magnus University, Studentu Str. 11, LT-53361 Akademija, Kaunas District, Lithuania; kestutis.romaneckas@vdu.lt (K.R.); ausra.sinkeviciene@vdu.lt (A.S.); 3 Department of Agricultural Engineering and Safety, Faculty of Engineering, Agriculture Academy, Vytautas Magnus University, Studentu Str. 15A, LT-53362 Akademija, Kaunas District, Lithuania; algirdas.jasinskas@vdu.lt

**Keywords:** *Vicia faba* L., multi-crop ash rates, sprouts, biometry, chlorophyll, dried matter

## Abstract

The use of burned plant biomass ashes could help not only with respect to utilizing combustion residues, but also with respect to optimizing the nutrition of cultivated agricultural plants without harming the environment. With this aim, a pot experiment of the effects of multi-crop biomass ash on faba bean seedlings was carried out in the Academy of Agriculture of the Vytautas Magnus University (VMU). Four ash fertilization rates were tested: 1. unfertilized (N0, comparative-control treatment); 2. fertilized at a low rate (N1, 200 kg ha^−1^); 3. fertilized at an average rate (N2, 1000 kg ha^−1^); 4. fertilized at a high rate (N3, 2000 kg ha^−1^). Final observations showed that ash fertilization significantly increases the height of faba bean sprouts by 21–38%, the length of the roots by 10–20% and the chlorophyll concentration in the leaves by 17%. The average green biomass of faba bean sprouts consistently increased with increasing fertilization rate, from 56% to 209%. Dried biomass increased by 160–220%. With increasing ash fertilization rate, the percentage of dry matter in the roots decreased by 10–50%. We recommend fertilizing faba bean with medium (1000 kg ha^−1^) and high (2000 kg ha^−1^) ash rates, as these rates led to the largest plants with the highest productivity potential.

## 1. Introduction

Biomass is one of the most important sources of renewable energy. It is projected that biomass combustion could provide between 33% and 50% of global energy demand by 2050 [1]. Currently, in Europe, the majority of biomass for bioenergy production comes from wood and forests, but it is predicted that the use of agricultural biomass and residues and waste will grow strongly in the future. It is expected that by 2050 more than half of the total biomass used for bioenergy could be agricultural biomass [2].

Wood usually has a relatively low ash content of between 0.3 and 5% by weight depending on the tree species, growing area or part of the tree, while bark, agricultural waste and other herbaceous fuels have higher ash content of up to 10% [3].

Calculating that 7 billion tons of biomass with an average ash yield on dry matter of 6.8% is burned annually for energy production, the amount of ash produced is around 476 million tons [4]. Large amounts of ash generated as a residue from the incineration process are considered a challenging solid waste worldwide. Ash production causes various environmental problems due to the large area required for its proper disposal and the toxic elements it contains. However, if a few decades ago fly ash was considered solid waste and the main cause of air, soil and water pollution, now it is increasingly recognized as a valuable material for various purposes [5].

During the biomass-combustion process, nitrogen (N) is mainly released in the flue gas, but other plant nutrients are present in high concentrations [6]. The main ash-forming elements are aluminium (Al), silicon (Si), calcium (Ca), iron (Fe), phosphorus (P), magnesium (Mg), sodium (Na) and potassium (K) [3]. Modern incinerators burn biomass to produce ash with low concentrations of heavy metals, making it suitable for agricultural use. Agricultural residue ash is rich in K and beneficial phosphates and could be suitable for fertilizer applications [7]. Biomass ash (BA) from biomass burning can be applied to soil as a sustainable recycling strategy for this waste, contributing to sustainable biomass production [8].

BA is free of pathogenic micro-organisms and has a number of comparative advantages compared to other potentially hazardous wastes (e.g., domestic sewage or sewage sludge) or by-products of agriculture or industry (manure, saturated sludge, digestate), which are either disposed of in a landfill site or spread on the soil without any prior conditioning [9].

BA is increasingly used in agriculture to improve soil properties. Ash improves soil electrical conductivity, water retention, organic carbon content and soil porosity and provides plants with essential nutrients. BA acts as a soil conditioner, improving the physical, chemical and biological properties of soil [5]. The use of untreated biomass fly ash as a soil improver or fertilizer is limited due to its high chemical reactivity and potentially toxic elements. Therefore, ash-based materials must be treated and stabilized before use. This not only reduces health and environmental risks, but also improves the function of BA as a soil conditioner [10]. The leaching of harmful elements from the ash is reduced by the natural ageing of the ash over a short period of time (1 month to 1 year). Environmental influences such as pH, redox potential, temperature, atmospheric humidity and CO_2_ can cause mineralogical, chemical and physical changes in ash [11].

When evaluating the properties of ash as a fertilizer, several aspects are taken into account, primarily with respect to nutrients with a mass fraction greater than 1%, to nutrient release rates and to total toxic elements [12].

When preparing ash for use as a fertilizer, it is important to consider not only the elemental composition of the ash, but also the soil in which it is to be applied. For example, low soil pH increases the availability of cadmium (Cd). The organic matter content is the second most important factor in determining Cd availability. High levels of organic matter can reduce Cd availability. In addition, organic matter improves the quality of ash as a fertilizer due to its potential mineralization and N availability. It is therefore recommended to choose soils with a high organic matter content [13].

Many studies have shown the positive effects of BA on soil and plants. Schönegger et al. [14] found that the application of fly ash had a positive effect on the chemical and microbiological properties of the soil, while no detrimental effects were recorded. The addition of fly ash resulted in an increase in soil pH, indicating that alkaline fly ash (pH = 12.5) can replace lime to reduce soil acidity to a level suitable for agriculture. Stanek-Tarkowska et al. [15] found that the addition of willow (*Salix viminalis* L.) biomass ash (K_2_O, 200 to 500 kg ha^−1^) to spring and black soil resulted in an increase in micro-organisms. A total of 44 bacterial species from 5 genera were identified. Ondrasek et al. [16] found that soil amendment with wood fly ash (0–10% w/w) resulted in a significant change in soil pHKCl (up to 9.1), an increase in salinity (>8.2-fold) and an increase in the content of most of the nutrients (up to 5.4-fold), but that the application of fly ash at a rate of more than 1.25% resulted in a reduction in the growth of the maize root and shoot, probably due to the effects of alkali stress. Boros-Lajszner et al. [17] found that maize (*Zea mays* L.) for energy purposes can be successfully grown in soil incorporated with ash from *Salix viminalis* biomass. It was found that even higher doses of ash did not deteriorate the calorific properties of corn. An ash rate of 5–10 g kg^−1^ soil dry weight did not impair either the growth or development of *Zea mays* L. However, a higher rate (20 g kg^−1^) of soil dry mass reduced the above-ground biomass of maize. It was also found that ash inhibited the activity of all analyzed soil enzymes, but increased soil pH and sorption capacity. Wang et al. [18] found that doses of 1%, 2.5% and 5% fly ash can increase the biomass and chlorophyll content of Chinese cabbage. Buneviciene et al. [19] found that fertilization with BA significantly increased grain and straw yields in spring barley. Ikeura et al. [20] found that ash from burning tomato pellets can be an effective fertilizer for growing vegetables, because the P, K, Ca and Mg content of this pellet ash is higher than that obtained from burning wood biofuel pellets. 

Despite the wealth of research, the use of BA in agriculture still raises many questions. The physical and chemical properties of ash can vary considerably depending on the form of the feedstock, the type of feedstock, the type of boiler and the firing temperature. Although biomass ash is used in agricultural and some forest soils, particularly in Europe, its use is limited by a number of barriers, including legal regulation, the cost of use, the variable quality of the ash and the uncertainty of the long-term effects on ecosystems [21]. Whether BA can be used as fertilizer in agriculture must be assessed on a case-by-case basis, depending on the origin of the biomass [22].

There is no single legal framework for the use of BA worldwide. Different countries have different legislation regulating the use of ash for fertilization. In Lithuania, only the use of wood biomass ash is defined. Some countries have not only maximum but also minimum concentrations for certain chemical elements. For example, Finland sets minimum concentrations for Ca, P and K in biomass ash used as fertilizer. The concentration of Ca in fly ash used in agriculture must be at least 10% and the total concentration of phosphorus and potassium must be at least 2% [23].

As biomass feedstocks have a wide variety of characteristics, and the properties of the ash from the combustion of different feedstocks also vary considerably, the potential for ash utilization needs to be investigated on a case-by-case basis. 

The authors have already carried out several studies supporting the suitability of multi-crop biomass for solid biofuel production [24,25,26]. This paper presents the results of a study to evaluate the suitability of ash obtained from the burning of biomass pellets of multi-crop plants (field beans, maize and fibrous hemp grown in the same field) for plant fertilization. 

The aim of this study was to establish the effect of burned multi-crop biomass pellet ash rates on faba bean germination and sprout development.

## 2. Results and Discussion

### 2.1. Biometry of the Sprouts

After the first measurement, it was found that the height of the field bean seedlings was 1.1 to 1.2 times lower in the pots with different rates of ash (Table 1). However, the opposite trends were found in the rest of the study period. When applying different fertilization rates, the height of faba bean sprouts was determined to be greater than that of the control treatment (N0). Similar results were found in our earlier investigations with spring barley [27]. Turp et al. [28] also found that using biomass power plant ash as an additive in cattle manure vermicompost stimulated bean growth.

During the first measurement, the longest roots (18.1 cm) were measured when fertilizing with the highest ash rate (N3) (Table 2). During the second measurement, it was found that when applying different ash rates, the roots of faba beans became significantly longer from 1.4 to 1.6 times compared to the control treatment. The data of the next two measurements varied.

Correlation data analysis showed average correlation between the rates of ashes and height of sprout canopy (r = 0.598; *p* > 0.05).

We found the average correlation between height of shoots and length of roots (r = 0.602; *p* > 0.05). Height of shoots also related with shoot and root dry matter percentage (r = 0.806; r = −0.658; *p* > 0.05) and shoot fresh and dried biomass (r = 0.759; r = 0.752; *p* > 0.05). 

### 2.2. The Chlorophyll Concentration in Faba Bean Leaves 

The concentration of chlorophyll in plant leaves usually depends on the conditions of irradiation and N nutrition. During ash fertilization, the plants did not receive additional mineral nitrogen, so the chlorophyll concentration in the leaves varied irregularly during the four measurements (Table 3). Cucci et al. [29] found the lowest chlorophyll index in unfertilized control, compared with other trials with some N editions. Similar results were found by other researches [28].

Fertilization rates increases are negatively correlated with chlorophyll concentration in leaves (r = −0.715; *p* > 0.05).

### 2.3. Sprout Biomass 

Studies have shown that in the first stages of faba bean sprout development, ash fertilization in most cases significantly reduced the percentage of dry matter in the shoots (Table 4). At the end of the experimentation, this influence became insignificant, although the percentage of dry matter increased as the fertilization rate increased. N3 treatment equaled 472 kg ha^−1^ of total K addition. Barłóg et al. [30] concluded that faba bean accumulated more dry matter in K-rich soil compared to K-poor soil. Sulfur (S) addition improved the crop growth rate.

Fertilization with ash usually did not significantly affect the percentage of dry matter in faba bean roots (Table 5). However, during the last measurement, it became clear that with increasing fertilization rate, the percentage of dry matter in roots significantly and consistently decreased, unlike faba bean shoots.

Generally, the rise of fertilization rate initiated the increase in dry matter percentage in the shoots (r = 0.951; *p* < 0.05) and, conversely, decreased percentage in the roots (r = −0.829; *p* > 0.05).

Conversely, in our experiment with spring barley, fertilization with ash usually reduced the amount of dry matter in barley shoots but increased it in their roots [27].

At the first development stages of faba bean sprouts, the average biomass of sprout was similar in all treatments and did not vary significantly (Table 6). During approximately a month from the first test, the fresh and dried biomass of shoots significantly increased with the increase in fertilization rate. N3 treatment equaled 54 kg ha^−1^ of total P addition. Xiao et al. [31] found that optimum P_2_O_5_-application rate for faba ban was approximately 62 kg ha^−1^. Lavrenko et al. [32] suggested the N_45_P_45_ fertilization formula. 

In our experiment with spring barley, treatment N2 was the most suitable [27].

The final results of experimentation showed a positive relation between fertilization rates and fresh or dried biomass of shoots (r = 0.860; 0.867; *p* > 0.05).

As in the evaluation of the biomasses of the faba bean shoots, the biomass of the roots did not differ significantly and varied depending on the irrigation regime (Table 7). At the end of the experiment, it was found that the highest fresh biomass of the sprout roots was in the average and high rates fertilized pots, but due to the decreasing of dry matter percentage, the dry biomass of the sprout roots was like that in the unfertilized pots. Kumar and Kumar [33] also found that fertilization with thermal power plant ash at a fertilization rate of 25 g m^−2^ increased legume yield.

Multi-crop ashes contain some S, which is important in faba bean nutrition. Pötzsch et al. [34] tested various S-containing fertilizers and did not find any significant differences in faba bean yields.

Fertilization rates were also related with the average fresh biomass of roots (r = 0.946; *p* > 0.05).

An earlier Müller-Stöver et al. [35] experiment showed that ash produced by low-temperature gasification of wheat straw had no effect on faba bean productivity, but increased barley and maize yields. The authors state that depending on the raw material, BA can replace mineral fertilizers, but it must be used taking into account the amount of nutrients, the needs of crops and the properties of the soil. These conclusions are confirmed by our study.

## 3. Materials and Methods

### 3.1. Site Description

A pot experiment was carried out in the Greenhouse of the Agriculture Academy (AA) of the Vytautas Magnus University (VMU). VMU AA is located near the second biggest town of Lithuania, Kaunas. 

### 3.2. Experimental Design and Agricultural Practice

The effect of multi-crop (maize, fibrous hemp and faba bean) biomass ash rates on faba bean (variety “Vertigo”) sprouts was addressed in a pot experiment in January and February 2024. Four ash rates were investigated:

1. Unfertilized (N0, comparative-control treatment).
2. Fertilized at a low rate (N1).
3. Fertilized at an average rate (N2).
4. Fertilized at a high rate (N3).

A pot experiment was performed with four replications. The area of the single pot was 0.05 m^2^. A technical substrate near neutral reaction was used. The substrate was mixed with Planosol soil in a ratio of 1:10. The soil was picked up in the fields of VMU Experimental Station. Soil pH was close to neutral, total nitrogen content was up to 0.175%, humus was 1.5–1.7%, mobile phosphorus was up to 323 mg kg^−1^, mobile potassium was up to 150 mg kg^−1^ and mobile magnesium was up to 506 mg kg^−1^.

**Figure 1:**
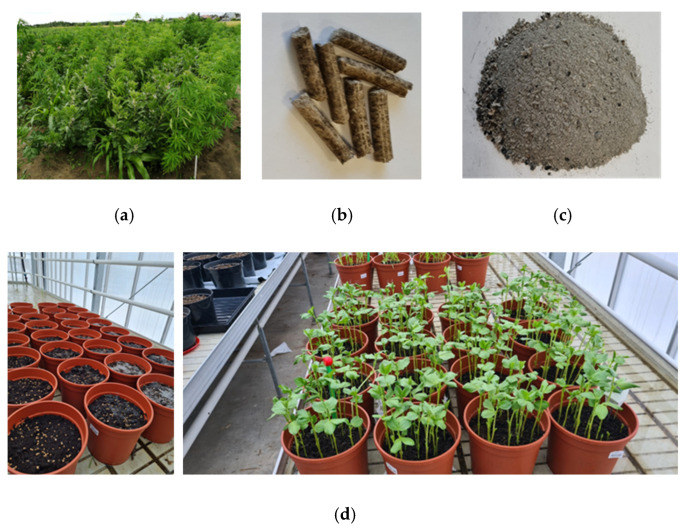
Stages of experimentation: (**a**) a multi-crop from which biomass is made into biofuel pellets; (**b**) samples of pellets from a multi-crop; (**c**) ash obtained after burning the pellets and used in the experiment; (**d**) study of the effect of ash on plants in a greenhouse.

After the filling of pots with the mix of substrate and soil, 7 g of ammonium sulfate nitrate (N_26_) or 140 g m^−2^ was incorporated into the pot layer of 0–5 cm depth. After, different rates of ashes were added: N1 at 1 g, N2 at 5 g, N3 at 10 g per pot (20, 100 and 200 g m^−2^ or 200, 1000 and 2000 kg ha^−1^, respectively). For example, in Austria, the maximum ash-application rate is exactly 2000 kg ha^−1^y^−1^. In Denmark, the application rate is 5 t ha^−1^ 5-y^−1^ [36]. The ash used for the experiment was obtained by burning biofuel pellets made from the biomass of maize, technical hemp and faba bean grown in the fields of VMU AA Experimental Station (Figure 1). Multi-crop growing technology is explained in more detail in Balandaitė et al. [37]. 

The ash used for the experiment is the ash from the laboratory combustion of biofuel pellets from the biomass of a multi-crop (maize, fibrous hemp and faba bean grown simultaneously in the same field in 2021). The biofuel pellets were also produced under laboratory conditions and data on their physico-mechanical properties and elemental composition have been reported in a previous publication [25] (see data for MIX3-1 variant).

The elemental composition of the ash was determined in the laboratories of the Lithuanian Energy Institute. Samples were mineralized according to ISO 16967:2015 [38] and LST EN ISO 16968:2015 [39] standards. The analysis of major elements was carried out in accordance with LST EN ISO 16967:2015 [38], and of minor elements in accordance with LST EN ISO 16968:2015 [39]. S and chlorine (Cl) analysis was carried out in accordance with standard LST EN ISO 16994:2016 [40]. The chemical composition of the ash from the combustion of solid biofuel pellets from multi-crop plants is presented in Table 8.

The dominant elements were Si, K, Ca, Mg and P. The concentrations of Cd, Cu, Pb and Zn in the ash did not exceed the limit values laid down in the Lithuanian, Polish, Finnish and Brazilian legislation governing the use of biomass ash for agricultural fertilization [1,23,41,42,43,44].

After adding the ashes, the surface of the pots was slightly compressed. After, 20 seeds per pot were placed on the top and covered with 3 cm substrate cover. In the greenhouse, the air temperature was approximately 18–20 °C, relative humidity was 50–60% and lighting time was up to 12 h. Irrigation was once a week to saturate the soil to its full moisture content.

### 3.3. Methods and Analysis

Ten faba bean sprouts without seeds of each pot were weighed for green biomass test. After, faba bean biomass samples were dried at a temperature of 105 °C in a drying oven (Memmert, Schwabach, Germany) to constant mass (LST ISO 751:2000 [45]). Seedling height and root length were also determined. Average plant height and root length (cm) were determined by measuring the plants in each experimental pot. Leaf chlorophyll concentration was measured with a chlorophyll meter MC-100 (Apogee Instruments, Logan, UT, USA). It measured chlorophyll concentration from red (653 nm) to short infrared (931 nm) wavelengths. At least 10 leaves of each plant per pot were measured.

Experimental data were processed using single factor analysis of variance (ANOVA) from the statistical software package SYSTAT, version 10 [46]. Significant differences between treatment and the control treatment were as follows: * *p* ≤ 0.050 > 0.010 (significant at 95% probability level), ** *p* ≤ 0.010 > 0.001 (significant at 99% probability level), and *** *p* ≤ 0.001 (significant at 99.99% probability level). 

A correlation analysis was applied to evaluate the causality of the studied traits. We used the program STAT ENG from the package ANOVA [47,48,49].

## 4. Conclusions

Faba bean-development indicators varied between observations, but the final observation showed that faba beans grown in ash-fertilized pots had significantly larger sprouts by 21–38%, 10–20% longer roots and 17% higher chlorophyll concentration in the leaves. 

Ash fertilization rates had little effect (2.5–6.2% points) on dry matter concentration in the leaves, but significantly decreased the concentration in the roots by 3–24%. Average green biomass of faba bean sprout consistently increased with increasing fertilization rate from 56 to 209%. Dried biomass increased by 160–220%. With increasing ash fertilization rate, the percentage of dry matter in the roots decreased by 10–50%. 

The use of ashes had a positive effect on faba bean development, although the effect was slow and was established at the last observation, a month from the beginning of experimentation. We recommend fertilizing faba bean with medium (1000 kg ha^−1^) and high (2000 kg ha^−1^) ash rates, as these rates grew the largest plants with the highest productivity potential.

## Figures and Tables

**Table 1:** The average height of faba bean sprout, cm.

Treatment/Date	22 January 2024	29 January 2024	5 February 2024	23 February 2024
N0	30.4	34.3	37.1	40.7
N1	28.2	36.8 *	40.4	55.3 ***
N2	28.1	34.8	41.3	49.5 **
N3	25.8 *	36.7 *	39.0	56.3 ***

Note: Unfertilized (N0), comparative-control treatment, fertilized at a low rate (N1), fertilized at an average rate (N2), fertilized at a high rate (N3); * significant differences at the 95% probability level, ** significant differences at the 99% probability level, *** significant differences at the 99.9% probability level.

**Table 2:** The average length of faba bean roots, cm.

Treatment/Date	22 January 2024	29 January 2024	5 February 2024	23 February 2024
N0	17.0	12.2	13.0	18.0
N1	16.4	19.3 ***	16.7 **	21.7 **
N2	13.4	16.8 ***	17.0 ***	16.2
N3	18.1	19.9 ***	11.2	19.9

Note: Unfertilized (N0), comparative-control treatment, fertilized at a low rate (N1), fertilized at an average rate (N2), fertilized at a high rate (N3); ** significant differences at the 99% probability level, *** significant differences at the 99.9% probability level.

**Table 3:** The chlorophyll concentration in faba bean leaves, µmol m^−2^.

Treatment/Date	22 January 2024	29 January 2024	5 February 2024	23 February 2024
N0	13.9	15.6	12.7	14.4
N1	15.2	12.4 *	11.8	16.4
N2	13.4	12.3 *	14.9 *	15.4
N3	13.4	12.0 *	14.9 *	12.6

Note: Unfertilized (N0), comparative-control treatment, fertilized at a low rate (N1), fertilized at an average rate (N2), fertilized at a high rate (N3); * significant differences at the 95% probability level.

**Table 4:** The percentage of dry matter in faba bean shoots.

Treatment/Date	22 January 2024	29 January 2024	5 February 2024	23 February 2024
N0	6.7	6.4	7.4	8.1
N1	7.2 **	6.2	6.4 ***	8.3
N2	6.1 **	4.8	6.6 ***	8.4
N3	6.1 **	6.6	6.7 ***	8.6

Note: Unfertilized (N0), comparative-control treatment, fertilized at a low rate (N1), fertilized at an average rate (N2), fertilized at a high rate (N3); ** significant differences at the 99% probability level, *** significant differences at the 99.9% probability level.

**Table 5:** The percentage of dry matter in faba bean roots.

Treatment/Date	22 January 2024	29 January 2024	5 February 2024	23 February 2024
N0	11.2	12.2	8.4	12.6
N1	12.1	10.2 **	8.5	11.3 *
N2	14.0	12.1	8.4	9.6 ***
N3	11.4	13.0	9.9	9.8 ***

Note: Unfertilized (N0), comparative-control treatment, fertilized at a low rate (N1), fertilized at an average rate (N2), fertilized at a high rate (N3); * significant differences at the 95% probability level, ** significant differences at the 99% probability level, *** significant differences at the 99.9% probability level.

**Table 6:** The average fresh and dried biomass of faba bean sprout shoot, g.

Treatment/Date	22 January 2024	29 January 2024	5 February 2024	23 February 2024
N0	3.37/0.21	3.34/0.20	3.21/0.24	2.48/0.20
N1	3.23/0.23	3.90/0.24	3.93/0.25	3.87 ***/0.32 ***
N2	3.38/0.21	3.90/0.19	4.05/0.27	4.97 ***/0.42 ***
N3	3.00/0.18	4.15/0.27 *	3.51/0.24	5.18 ***/0.44 ***

Note: Unfertilized (N0), comparative-control treatment, fertilized at a low rate (N1), fertilized at an average rate (N2), fertilized at a high rate (N3); * significant differences at the 95% probability level, *** significant differences at the 99.9% probability level.

**Table 7:** The average fresh and dried biomass of faba bean sprout roots, g.

Treatment/Date	22 January 2024	29 January 2024	5 February 2024	23 February 2024
N0	0.82/0.09	0.42/0.05	0.33/0.03	0.55/0.10
N1	0.96/0.12	0.90 **/0.09 *	0.64/0.05	0.42/0.05 *
N2	0.86/0.12	0.45/0.05	0.65 */0.05	0.70/0.07
N3	0.69/0.08	0.63/0.08	0.44/0.04	0.94 */0.09

Note: Unfertilized (N0), comparative-control treatment, fertilized at a low rate (N1), fertilized at an average rate (N2), fertilized at a high rate (N3); * significant differences at the 95% probability level, ** significant differences at the 99% probability level.

**Table 8:** Ash chemical composition.

Chemical Element	Amount	Chemical Element	Amount
Ca, mg kg^−1^	123,449.73 ± 6.22	Al, mg kg^−1^	3741.99 ± 12.44
Cd, mg kg^−1^	<0.51	K, mg kg^−1^	236,102.69 ± 16.36
Cu, mg kg^−1^	81.80 ± 10.16	Na, mg kg^−1^	9383.18 ± 12.04
Fe, mg kg^−1^	4512.95 ± 10.02	P, mg kg^−1^	27,063.62 ± 5.82
Mg, mg kg^−1^	34,676.88 ± 12.22	Si, mg kg^−1^	276,629.95 ± 5.68
Pb, mg kg^−1^	<1.20	Cl, %	2.98 ± 0.10
Zn, mg kg^−1^	265.02 ± 5.71	S, %	1.36 ± 0.07

## Data Availability

Data is contained within the article.
